# “Immunolocalization and effect of low concentrations of Insulin like growth factor‐1 (IGF‐1) in the canine ovary”

**DOI:** 10.1002/vms3.347

**Published:** 2020-09-07

**Authors:** Diogo J. Cardilli, Kellen Sousa‐Oliveira, Carolina Franchi‐João, Faviana Azevedo‐Voorwald, Marco A. Machado‐Silva, João Ademir Oliveira, María Jesús Sánchez‐Calabuig, Gilson H. Toniollo, José F. Pérez‐Gutiérrez

**Affiliations:** ^1^ Departamento Medicina y Cirugía Animal Facultad de Veterinaria Universidad Complutense de Madrid Madrid Spain; ^2^ Departamento de Zootecnia. Escola de Veterinária e Zootecnia Universidade Federal de Goiás Goiânia‐GO Brazil; ^3^ Instituto de Medicina Veterinária da Universidade Federal do Pará Castanhal‐PA Brazil; ^4^ Faculdade de Ciências Agrârias e Veterinârias Universidade Estadual Paulista Jaboticabal Brazil; ^5^ Departamento de Medicina Veterinária. Escola de Veterinária e Zootecnia Universidade Federal de Goiás Goiânia‐GO Brazil; ^6^ Departamento de Ciências Exatas da Faculdade de Ciências Agrárias e Veterinárias Faculdade de CiênciasAgrárias e Veterinárias Universidade Estadual Paulista Jaboticabal Brazil; ^7^ Departamento de Medicina Veterinária Preventiva e Reprodução Animal Faculdade de CiênciasAgrárias e Veterinárias da Universidade Estadual Paulista Jaboticabal Brazil; ^8^ Present address: Ministério da Agricultura Pecuária e Abastecimento Departamento de inspeção de produtos de origem animal Unidade Técnica Regional de Uberlândia‐MG Brazil

**Keywords:** bitch, insulin like growth factor‐1, maturation, oocyte

## Abstract

Insulin like growth factor‐1 (IGF‐1) plays an important role in the regulation of ovarian function. Despite its extensive study in several species, there is a paucity of information about IGF‐1`s function and localization in the canine ovary. The aim of the present study was to assess the effect of IGF‐1 on oocyte nuclear maturation and to immunolocalize the IGF‐1 and its receptor (IGF‐1R) in the ovary. Cumulus‐oocyte complexes (COCs) were obtained from 34 bitches. The COCs from each bitch were incubated in TCM 199‐HEPES in the absence (*n* = 199) or presence (*n* = 204) of 100 ng/ml IGF‐1 for 96 hr at 38ºC in 5% CO_2_, stained and evaluated for nuclear maturation by fluorescence microscopy. The results showed that the addition of IGF‐1 did not have an effect (*p* ˃ 0.05) on the nuclear maturation under these conditions. The immunohistochemical study revealed nuclear and cytoplasmic staining for IGF‐1 and IGF‐1R, respectively. Both were localized in all ovarian structures including the corpus luteum, but not in the granulosa cells from primordial follicles. In addition, IGF‐1 was not localized in the oocytes in tertiary follicles. The results obtained show the presence of IGF‐1 through the stages of follicular growth and in the corpus luteum of the canine ovary. However, its role on oocyte nuclear maturation could not be demonstrated.

## Graphical abstract

The Insulin like growth factor‐1 (IGF‐1) was localized by immunohistochemistry in the canine ovary from 34 bitches. IGF‐1 and IGF‐1R were localized in all ovarian structures including the corpus luteum, but not in the follicular cells from primordial follicles in the canine ovary. The results showed that the addition of IGF‐1 at 100 ng/ml did not have effect (*p* ˃ 0.05) on the nuclear maturation.

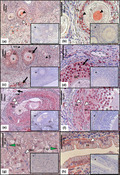

## 1 INTRODUCTION

The development of assisted reproductive technologies (ART) in domestic canids arouses great interest due to its critical role in conserving endangered canid species. Additionally, as dogs are exposed to the same environmental factors as their human companions, advanced reproductive techniques in dogs may also be utilized as a translational biomedical research model. Despite multiple studies, its full potential has not yet been achieved due to difficulties attaining the in vitro maturation of the oocytes (Nagashima et al., 2015).

One drawback is the specific requirements of the canine oocyte, which impairs the use of protocols that have been successful in other species. In the bitch, ovulation takes place in a progesterone‐dominated environment and the oocyte is ovulated in the immature germinal vesicle stage, at the beginning of the first meiotic division (prophase I stage). The oocyte completes meiosis after a prolonged exposure (i.e. 2–3 days) to the oviductal environment (Songsasen & Wildt, 2007), which consists of a changing milieu in response to multiple fluctuating hormones throughout the oestrous cycle. Therefore, this environment is difficult to reproduce in vitro and involves the complex interaction of a multitude of signals that likewise vary throughout the oestrous cycle (Luvoni, Chigioni, Allievi, & Macis, 2005).

The domestic dog is a non‐seasonal, polytocous and spontaneous ovulator species. The bitch enters oestrous once or twice a year, at 5–12 months intervals. The canine cycle shows uncommon features. Its long pro‐estrous can last for 3 days to 3 weeks, with an average of 1 week, and it is characterized by high oestrogen (E2) levels, responsible for the swelling of the vulva, congestion of the reproductive tract, and a bloody vaginal discharge. Pro‐oestrous is followed by oestrous, a period of receptivity to mating, which lasts 3–21 days or even more, and it is accompanied by a decrease in E2 and an increase in progesterone (P4). Oestrous is followed by dioestrous, a P4 dominated phase that lasts an average of 2 months in the non‐pregnant bitch. Towards the end of dioestrous, progesterone declines as the bitch enters a long lasting anoestrous of 2 to 10 months, characterized by lacking gonadal activity (Concannon, McCann, & Temple, 1989).

In most mammals, basal follicular growth is mainly modulated by FSH, whereas terminal follicular growth and ovulation depend on both FSH and LH. However, the granulosa cells of the bitch acquire LH receptors very early compared to other species (Chastant‐Maillard et al., 2011).The LH peak rises during oestrous, about 8 days before the onset of dioestrous and it is associated to decreased levels of E2 and increased P4 concentration (Concannon, Hansel, & Visek, 1975; Holst & Phemister, 1975).

In many mammals, the LH surge triggers meiotic resumption by interrupting communication through GAP junctions between the cumulus cells and the oocyte, thus preventing the transfer of inhibitory signals to the oocyte (Raman 2001). However, in the canine oocyte, the cumulus cells remain tightly bound to the oocyte for several days after the LH peak and ovulation (Reynaud et al., 2006). The canine LH surge is quite long, 1–5 days (Wildt, Panko, Chakraborty, & Seager, 1979), compared to other species, such as the cow, that last only 8 hr (Dieleman, Bevers, Poortman, & van Tol, 1983). The time lapse between the LH surge and ovulation in the bitch is 48 hr, while the time between the LH surge and the meiotic resumption is extended for about 5 days (Reynaud et al., 2006). Maturation takes place in the oviduct. At ovulation, immature oocytes are expelled from the ovarian bursa together with 1–2 ml of follicular fluid, containing among other factors, Insulin like growth factor‐1 (IGF‐1) (Balogh, Muller, Boos, Kowalewski, & Reichler, 2018; Chastant‐Maillard et al., 2011).

Studies in other species (i.e., rats, mice, sheep, bovine, primates and human) have shown that IGF‐1 plays a central role in the integration of these signals, because it can interact with gonadotropins and steroids, known regulators of oocyte physiology (Lackey, Gray, & Henricks, 1999). In the bovine granulosa cells IGF‐1 receptor (IGF‐1R) has been shown to increase the FSH receptors and the LH receptors as well as, mRNA expression of the steroidogenic genes: *CYP11A1, HSD3B1* and *CYP19A1 (*Mani et al., 2010; Rawan, Yoshioka, Abe, & Acosta, 2015 *)*. In the follicle IGF‐1 bioavailability is regulated by IGF‐1 binding proteins (Ui, Shimonaka, Shimasaki, & Ling, 1989). Once free, IGF‐1 acts through an autocrine/paracrine mode on the granulosa cells and oocyte, regulating cell proliferation, differentiation, survival and steroidogenesis as well as oocyte maturation (Mazerbourg, Bondy, Zhou, & Monget, 2003). Plasma and intrafollicular IGF‐1 levels from antral follicles during the follicular phase in the dog correlate significantly (Reynaud, Chastant‐Maillard, Batard, Thoumire, & Monget, 2010) so endocrine effects of IGF‐1 cannot be excluded.

The effects of IGF‐1 are mediated through the type I IGF‐1 receptor (IGF‐1R), a transmembrane tyrosine kinase receptor, by activating the phosphoinositide 3´‐OH kinase (PI3kinase)/Akt pathway, involved in cell survival, and the MAPK pathways implicated in cell proliferation and differentiation e.g., luteinization of follicular cells by enhanced StAR protein expression (Sasaoka et al., 1996; Sekar, Lavoie, & Veldhuis, 2000).

In addition, both pathways are involved in oocyte maturation through the activation of the maturation‐promoting factor (MPF) that facilitates the entry into M‐phase of meiosis I and II (Kishimoto, 2003). It has been demonstrated that insulin like growth factor promotes maturation and expansion of cumulus cells (Mazerbourg et al., 2003), and can also inhibit apoptosis in ovarian cells (Solomon‐Zemler, Sarfstein, & Werner, 2017; Wasielak & Bogacki, 2007).

Information regarding the participation of IGF‐1 in meiosis were obtained from different species (Mazerbourg et al., 2003). However, despite the applicative interest, knowledge about the role of the IGF‐1 system in the reproductive physiology of canids remains sparse.

The presence of IGF‐1 and IGF‐1R has been detected in the follicular and luteal cells in the canine ovary (Almeida et al., 2015; Balogh et al., 2018), suggesting that it could play a role on gamete physiology regulation. In this regard, a recent study by (Sato et al., 2018) found beneficial effects of a short incubation time (48 hr) and high IGF‐1 concentrations (50 µg/ml) on oocyte maturation, suggesting the IGF‐1R presence on the immature oocyte.

However, despite the beneficial effects, it is possible that high IGF‐1 concentrations will have a toxic effect or down regulate IGF‐1R on the oocyte (Lorenzo, Illera, Illera, & Illera, 1994; Rechler & Nissley, 1985). Thus, utilizing a lower concentration for a longer incubation may improve results.

The aim of the current study is to reveal the immunolocalization of IGF‐1 and IGF‐1R in all the canine ovarian compartments throughout the cycle and to assess the effects of low IGF‐1 concentration (100 ng/ml) on nuclear maturation using a long incubation time (96 hr).

## 2 MATERIAL AND METHODS

All media and chemicals were obtained from Sigma‐Aldrich Chemical (St Louis, MO, USA) unless otherwise indicated.

### 2.1 In vitro maturation

Ovaries from 34 bitches of different breeds and ages (8 months to 7 years old), were collected after elective ovariohysterectomy at Veterinary Teaching Hospital of Sao Paulo State University (FCAV‐UNESP, Jaboticabal, Brazil). The stage of the oestrous cycle was determined before surgery, by vaginal cytology and blood serum progesterone concentration through the radioimmunoassay kit Coat‐a‐count® (Diagnostic Product Corp., Los Angeles, CA, USA). The stage was then later confirmed by macroscopic characterization of the ovaries (Otoi, Ooka, Murakami, Karja, & Suzuki, 2001).

Ovaries were removed, washed, released from the ovarian bursa and maintained for 5–10 min in physiological saline (0.9% NaCl) at 37ºC. Then ovaries were placed in sterile containers in PBS + 10% foetal calf serum (FCS) at 37ºC and transferred to Petri plates containing the same solution, where they were sliced (2 mm thick) to release the cumulus‐oocytes complexes (COCs).

Oocytes were washed in a previously filtered (0.22 µm sterile filter) TCM 199‐HEPES solution supplemented with 10% FCS, 0.2 mM sodium pyruvate and 83.35 µg/ml amikacin sulfate solution at 37ºC and classified according to Hewitt and England (Hewitt & England, 1998).

A total of 403 grade I oocytes were used (12 from pro‐oestrous, 95 from oestrous, 142 from dioestrous and 154 from anoestrous bitches). All the grade I oocytes that exhibited a dark uniform ooplasm, an intact zona pellucida, a diameter greater than 100 µm and were surrounded by one or more layers of cumulus cells, were selected for this study.

A maximum of 10 randomly chosen COCs from the same donor were cultured per plate in either: 500 μL of TCM 199 sodium bicarbonate supplemented with 10% FCS, 0.2 mM sodium pyruvate, 83.35 µg/ml amikacin sulfate, 1 µg/ml FSH, 5 µg/ml hCG, 1 µg/ml 17β‐estradiol solution (TCM‐C, *n* = 199) or 500 μL of TCM‐C supplemented with 100 ng/ml of IGF‐1 (TCM‐I, *n* = 204). Plates were incubated at 38.5ºC in a humidified 5% CO_2_ atmosphere, for 96 hr (Apparicio et al., 2011).

After incubation, cumulus cells were removed from the oocytes by gently pipetting in PBS containing 0.2% hyaluronidase for 5 min. After removing the cumulus cells, hyaluronidase was inactivated by washing the denuded oocytes in a PBS + 10% FCS solution. Then oocytes were kept for 1 hr in a solution containing Triton X‐100 in 0.3% BSA + PBS, fixed and stained with HOESCHT (Bisbenzimide H33342 Fluorochrome) in glycerol. The nuclear maturation was evaluated through an epifluorescence microscope (Axiovert 100, Carl Zeiss Inc., Oberkochem, Germany) with a 365–480 nm filter, according to Hewitt and England (Hewitt & England, 1998).

### 2.2 Immunohistochemistry

Ovarian samples from 34 bitches [anoestrous (*n* = 13), dioestrous (*n* = 12), oestrous (*n* = 8) and pro‐oestrous (*n* = 1)] were fixed and embedded in paraffin at the Veterinary Pathology Laboratory at the FCAV‐UNESP. Sections 3 µm thick were cut and mounted on poly‐L‐lysine slides, deparaffinized in xylenes and rehydrated in graded ethanol solutions. In order to retrieve the antigens, sections were immersed in 0.58% sodium citrate buffer (pH 6) and heated in a pressure cooker until reaching the boiling temperature. Then the cooker was closed to increase the pressure for 10 min. After cooling for 30 min at 4ºC, sections were rinsed in distilled water for 5 min and immersed in 3% hydrogen peroxide/methanol solution for 30 min to quench endogenous tissue peroxidases. The slides were then rinsed in PBT buffer (6 mM phosphate, 140 mM chloride, 4.2 mM potassium, 146 mM sodium, 0.3% (v/v) Triton X‐100, pH 7.2–7.4) and preincubated in blocking buffer containing 5% normal rabbit serum for 30 min at room temperature to prevent non‐specific reactions.

Incubation with the primary antibody was conducted overnight in a humidified chamber at 4ºC using the following antibodies: anti‐IGF‐1 (Cat.# PC195L, Calbiochem, La Jolla, CA, USA) and anti‐IGF‐1R (Cat.# PC196L, Calbiochem), both goat polyclonal antibodies diluted to 10 µg/ml and 5 µg/ml in PBT, respectively.

Afterwards, incubated sections were washed in PBT for 5 min. Antibody binding was detected using Vectastatin ABC kit (Vector Laboratories, Burlingame, CA, USA) adding rabbit anti‐goat biotinylated IgG (Cat.# 28,171, Anespec, San José, CA, USA) at 10 µg/ml in PBT, incubated for 30 min at room temperature, and followed by the addition of an avidin‐biotin‐peroxidase conjugate (Elite ABC Kit, Vector Laboratories, Burlingame, CA, USA). The reaction was revealed with 3‐amino‐9‐ethylcarbanzole (AEC, Vector Laboratories), which produces a red staining upon reaction with peroxidase, counterstained with Carazzi hematoxylin (Panreac, Barcelona, Spain) for 10 s and rinsed in water. Finally, sections were cover‐slipped in the aqueous‐based mounting medium Aquatex® (Merck, Darmstadt, Germany) and evaluated under the microscope.

Positive and negative controls were included in each experiment. Positive controls for IGF‐1 consisted of canine endometrial glands from the uterus with histologically diagnosed cystic endometrial hyperplasia (CEH) (De Cock, Ducatelle, Tilmant, & De Schepper, 2002) and mice testes (C57BL/6) for IGF1‐R (Pitetti et al., 2013). Both tissues know how to express IGF‐1 and IGF‐1R. Negative controls were performed in all samples by incubating with buffered phosphate solution (pH 7.2) or blocking buffer (i.e. normal goat serum) instead of primary antibody.

### 2.3 Statistical analysis

The statistical assessment for the in vitro maturation and analysis of the data were performed using the Chi‐square test (comparisons between groups and among groups at the varying phases of oestral cycle). When expected frequencies were lower than 5, Fisher's exact test were performed. Both tests were carried out using the statistical software SAS 9.1 (SAS Institute, Cary, NC, USA).

## 3 RESULTS

### 3.1 In vitro maturation

A total of 403 grade 1 COCs were obtained from 34 bitches at different stages of the oestrous cycle. The majority of the COCs were obtained from bitches that were in anoestrous (*n* = 13) and dioestrous (*n* = 12), rather than in oestrus (*n* = 8) or pro‐oestrus (*n* = 1).

After their recovery from the ovary, COCs were incubated in TCM 199 (TCM‐C) or TCM 199 supplemented with 100 ng/ml IGF‐1 (TCM‐I) for 96 hr, cumulus cells were removed and oocytes were evaluated for nuclear status. One hundred twenty seven oocytes were lost performing the procedure, specifically in the process of pipetting, removing the cumulus cells and staining. The remaining 276 oocytes were evaluated.

The results, expressed as absolute and relative frequencies of the stages of meiotic development, showed that IGF‐1 did not have an effect (*p* > .05) on the nuclear maturation of grade I canine oocytes (Table 1) regardless the stage of the oestrous cycle of the donor bitch (*p* > .05), (Table 2). The rate of oocytes that had resumed meiosis was not significantly different between groups (28.16% TCM‐C versus. 20.17% TCM‐I; *p* > .05). No oocyte matured up to metaphase II stage in either group (Tables 1 and 2).

**TABLE 1:** Effect of IGF‐1 on the nuclear maturation of canine oocytes

Meiotic stage	Absolute and relative (%) frequencies	
	TCM‐C	TCM‐I
GV	7 (5.04)^a^	8 (5.84)^a^
GVBD	28 (20.24)^a^	23 (16.55)^a^
MI	11 (7.82)^a^	5 (3.62)^a^
MII	‐	‐
Deg	93 (66.90)^a^	101 (73.99)^a^
Total	139 (100)	137 (100)

Absolute and relative frequencies of the stages of meiotic development of grade I canine cumulus‐oocyte complexes incubated in 500 μL of TCM 199 sodium bicarbonate supplemented with 10% FCS, 0.2 mM sodium pyruvate, 83.35 µg/ml amikacin sulfate, 1 µg/ml FSH, 5 µg/ml hCG, 1 µg/ml 17β‐estradiol solution (TCM‐C) or in 500 μL of TCM‐C supplemented with 100 ng/ml IGF‐1 (TCM‐I) for 96 hr. Germinal vesicle (GV), germinal vesicle breakdown (GVBD), metaphase I (MI), metaphase II (MII) and degenerating oocytes (Deg). Values followed by the same letter within the same row are not significantly different (*p *˂ 0.05).

**TABLE 2:** Effect of IGF‐1 on the nuclear maturation of canine oocytes obtained at different stages of the oestrous cycle

Meiotic stage	Stage of the oestrous cycle					
	Anoestrous		Dioestrous		Oestrous	
	TCM‐C	TCM‐I	TCM‐C	TCM‐I	TCM‐C	TCM‐I
GV	4^a^	6^a^	2^a^	2^a^	1^a^	0^a^
GVBD	13^a^	13^a^	12^a^	7^a^	3^a^	3^a^
MI	3^a^	1^a^	4^a^	2^a^	4^a^	2^a^
MII	—	—	—	—	—	—
Deg	44^a^	41^a^	33^a^	44^a^	16^a^	6^a^
TOTAL	**64**	**61**	**51**	**55**	**24**	**21**

Absolute frequencies of the stages of meiotic development of grade I COCs obtained from bitches at anoestrus (*n* = 13), dioestrous (*n* = 12) and oestrous (*n* = 8). Nuclear maturation was evaluated after 96 hr of incubation in 500 μL of TCM 199 sodium bicarbonate supplemented with 10% FCS, 0.2 mM sodium pyruvate, 83.35 µg/ml amikacin sulfate, 1 µg/ml FSH, 5 µg/ml hCG, 1 µg/ml 17β‐estradiol solution (TCM‐C) or in 500 μL of TCM‐C supplemented with 100 ng/ml IGF‐1 (TCM‐I).

Germinal vesicle (GV), germinal vesicle break down (GVBD), metaphase I (MI), metaphase II (MII) and degenerating oocytes (Deg). Values followed by the same letter within the same row are not significantly different (*P *˂ 0.05).

### 3.2 Immunolocalization of IGF‐1 and IGF‐1R

The immunostaing of IGF‐1 and IGF‐1R was evaluated subjectively in the following cell groups: vascular wall, in the layer of endothelial cells that covers all blood vessels; ovarian superficial epithelium, in the layer of simple squamous to cuboidal epithelium that covers the ovary; cortical tubules, particularly the cystic like structures present in the stroma, near the ovarian superficial epithelium, which are thought to be invaginations of the superficial epithelium; granulosa cell cords, specifically the endocrine cells that are similar to granulosa cells, probably derived from atretic follicles; ovarian stroma, particularly the stromal spindle‐shaped cells; granulosa cells at different stages of development from primordial, primary, secondary and tertiary follicles; oocytes at different stages of development from primordial, primary, secondary and the tertiary follicle. Finally, we also evaluated IGF‐1 and its receptor in the theca cells and luteal cells (granulosa and theca lutein cells).

**FIGURE 1:**
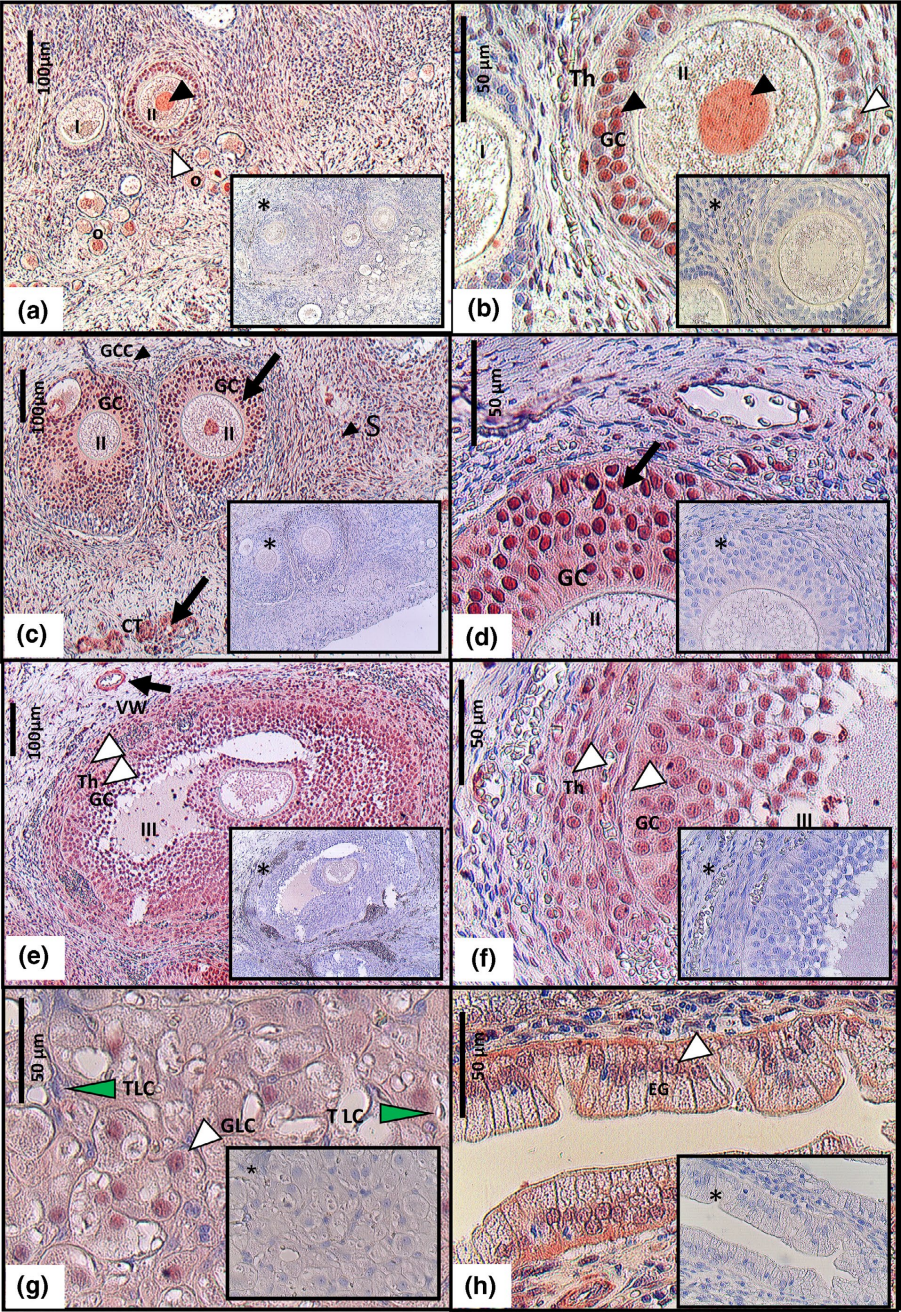
Immunohistochemical localization of IGF‐1 in the canine ovary (a – g). Immunoreactivity was detected in the nuclei and weakly in the cytoplasm of: granulosa cells (GC), theca cells (Th), granulosa cell cords (GCC), granulosa lutein cells (GTC), cortical tubules (CT), spindle shaped cells in the stroma (S), vascular wall (VW), as well as in the nuclei of oocytes in primordial (O), primary (I) and secondary (II) follicles. Black arrows indicate strong positive staining, black arrowheads indicate moderate staining, white arrowheads indicate weak positive staining and green arrowheads indicate a negative/very weak staining of the theca lutein cells (TLC). Note that TLC showed no nuclear staining. Negative staining were found in granulosa cells from primordial follicles and in oocytes from tertiary follicles (III). Positive control (h), endometrial glands (EG) of canine uterus that presented cystic endometrial hyperplasia. Negative controls (*)

**FIGURE 2:**
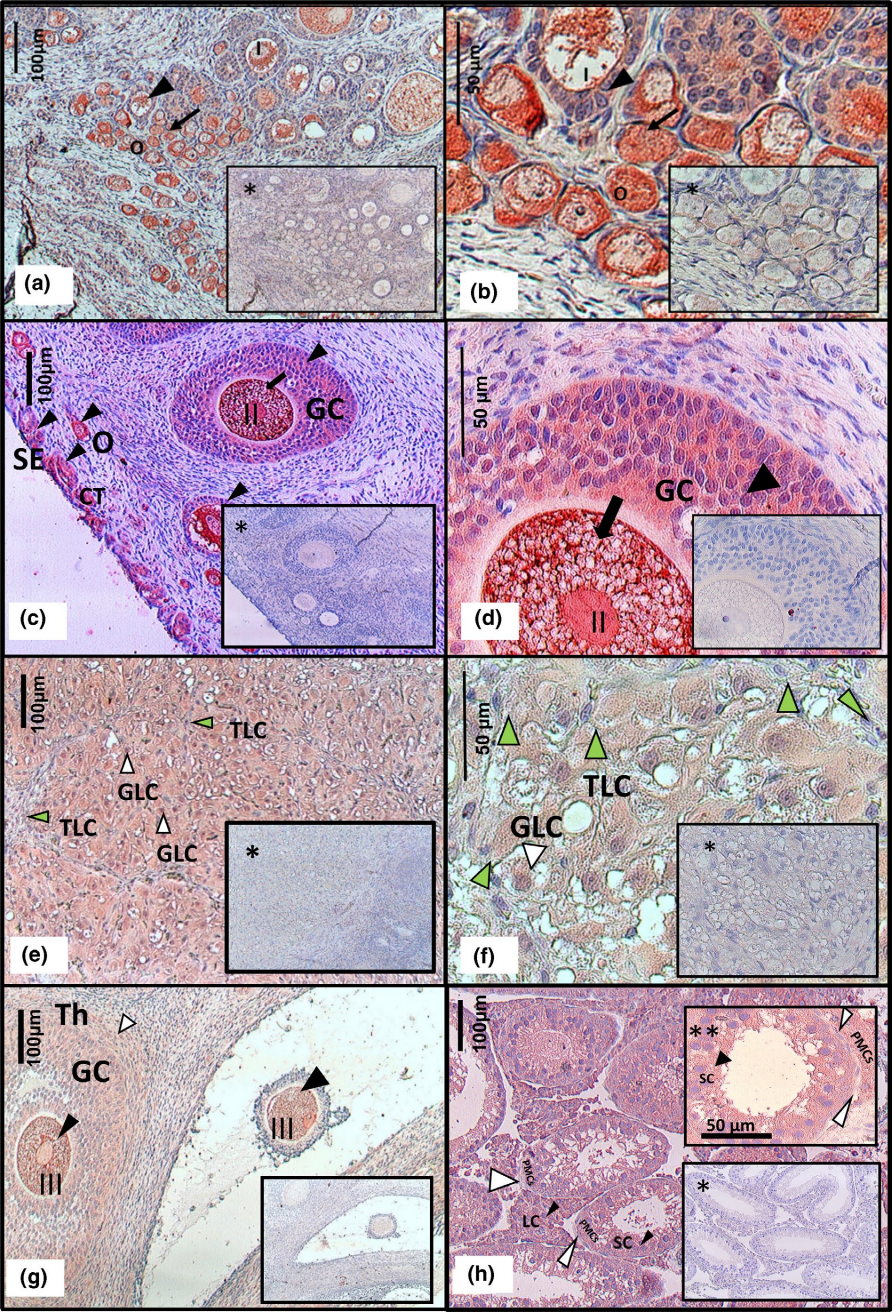
Immunohistochemical localization of IGF‐1R in the canine ovary (a –g). Immunoreactivity was detected in the cytoplasm and to a lower degree in the nuclei of: granulosa cells (GC), theca cells (Th), granulosa cell cord (GCC), granulosa lutein cells (GTC), cortical tubules (CT) and ovarian superficial epithelium (SE), as well as in the nuclei of oocytes in primordial (O), primary (I), secondary (II) and tertiary follicles (III). Black arrows indicate strong positive staining, black arrowheads indicate moderate staining, white arrowheads indicate weak positive staining and green arrowheads indicate negative/very weak staining of the theca lutein cells (TLC). Note that TLC show of no nuclear staining. Positive control (h), mice testis moderate staining was observed in testis in the Leydig cells (LC), and Sertolli cells (SC) weak staining in the peritubular myoid cells (PMCs). Higher magnification showing PMCs weak staining (**). Negative controls (*)

Immunohistochemistry revealed the presence of the IGF‐1 and IGF‐1R in the ovary of the bitch at the interstitial and follicular compartments, as well as in the corpus luteum (Table 3, Figures 1 and 2). Inside the cell, IGF‐1 was localized in the nucleus and it was weakly detectable in the cytoplasm (Figure 1) whereas IGF‐1R staining was primarily cytoplasmic and to a lower degree in the nucleus (Figure 2). All groups expressed both proteins (vascular wall; cortical tubules; granulosa cell cords: ovarian stroma; superficial epithelium; oocytes from primordial follicles; granulosa cell and oocytes from primary follicles; granulosa, theca cells and oocytes from secondary follicles, granulosa and theca cells from tertiary follicles; granulosa luteinized cells and theca luteinized cells but note that did not show nuclear staining). Granulosa cells from primordial oocytes, did not stain for the IGF‐1 or IGF‐1R (Table 3, Figures 1a, 2a,b). Oocytes from tertiary follicles were only immunoreactive for IGF‐1R (Table 3, Figure 2g).

**TABLE 3:** Relative intensity for immunohistochemical staining for IGF‐1 and for IGF‐1R in the ovaries of the bitch

Ovarian compartments	Type of cells	IGF‐1	IGF‐1R
Stroma	Superfical epithelium	++	++
	Cortical Tubules	+++	++
	Granulosa cell cords	++	++
	Spindle shaped cells	++	+
	Vascular wall	+++	+++
Follicles			
Primordial	Granulosa cells	**‐**	**‐**
	Oocyte	+	+++
Primary	Granulosa Cells	+	++
	Oocyte	++	+++
Secondary	Theca Cells	++	+
	Granulosa Cells	+++	+++
	Oocyte	++	+++
Tertiary	Theca Cells	++	+
	Granulosa Cells	++	++
	Oocyte	**‐**	++
Corpus Luteum	Theca lutein Cells	−/+	−/+
	Granulosa lutein Cells	+	+

Indicate strong staining (+++), moderate staining (++), weak staining (+), negative/very weak positive staining (−/+), negative (‐).

The highest level of expression for IGF‐1 were observed in the vascular wall and in the granulosa cells from the secondary follicle (Figure 1d) whereas for IGF‐1R the highest level of expression were observed in the oocyte until the tertiary stage of development (Table 3, Figure 2c and d).

Immunoreactivity was clearly detected in the endometrial glands of canine CEH uterus (Figure 2h) and in the seminiferous tubule of mice testis, (Figure 4h), used as positive controls for IGF‐1 and IGF‐1R, respectively. No immunoreactivity was observed in the negative control sections (Figures 2 and 4, insets*), i.e. the same sections with no staining.

## 4 DISCUSSION

### 4.1 In vitro maturation

Insulin like growth factor‐1 may have a positive effect on the maturation of canine oocytes. Recently, (Sato et al., 2018) improved the maturation rates to MII stage using a concentration of 50 µg/ml IGF‐1 and an incubation time of 48 hr. We investigated the use of a 500 times lower concentration of IGF‐1 in concert with an extended incubation time of 96 hr, both of which are closer to in vivo parameters (Evans, 1933). However, contrary with expected, no difference was observed in the nuclear maturation rate of oocytes or the rate of meiosis between IGF‐1‐treated and non‐treated groups. This may be explained by various factors including the stage of the reproductive cycle. Some authors (Kim et al., 2004; Luvoni, Luciano, Modina, & Gandolfi, 2001; Otoi et al., 2001; Willingham‐Rocky, Hinrichs, Westhusin, & Kraemer, 2003; Yamada et al., 1993) have suggested that the oocytes obtained from pro‐oestrous or oestrous bitches are more likely to reach MII stage compared to those of anoestrous or dioestrous females. From the 34 bitches used in our experiment, only 8 were in oestrous and 1 in pro‐oestrous, which may have influenced the negative result. In addition it could be explained by the low concentrations of IGF‐1 (100 ng/ml), which are high enough to mature porcine (Xia, Tekpetey, & Armstrong, 1994) and bovine (Lorenzo et al., 1994) oocytes, may be too low to reach the MII stage in the canine oocyte. It is therefore possible that the use of higher concentrations is more useful to mature canine oocytes, even though there are potential drawbacks, such as toxic effects of higher IGF‐1 concentrations and, as suggested by (Rechler & Nissley, 1985), down regulation of IGF‐1R.

### 4.2 Immunolocalization

The possibility that IGF‐1 exerts its biological effect on canine ovary is in agreement with the IGF‐1R localization in the different ovarian cell group, studied in the present study. Results from the immunohistochemical study are in consonance with the only previous study that detected IGF‐1 and IGF‐1R in the canine follicular and luteal cells (Balogh et al., 2018). However, the present study is the first to reveal the presence of both IGF‐1 and IGF‐1R within the ovarian granulosa cell cords, ovarian stroma, cortical tubules and superficial epithelium in the bitch. These results differ from those observed in pigs, where staining was restricted to the theca, granulosa and luteal cells, as well as the rat, where IGF‐1 was confined to the granulosa cells, suggesting a species‐specific pattern of expression (Mazerbourg et al., 2003).

In general these results are in agreement with those obtained in the sheep (Monte et al., 2019) and in the bitch ovary (Balogh et al., 2018; Sato et al., 2018). The localization of IGF‐1 and IGF‐1R through the different stages of follicular development suggests a change from an autocrine to a paracrine action as the follicle grows. In the primordial follicle, IGF‐1 and IGF‐1R were both detected in the oocyte but not in the follicular cells suggesting an autocrine role during follicular recruitment. However, during secondary stages of follicular development, both granulosa cells and the oocyte expressed IGF‐1 and IGF‐1R, suggesting a autocrine/paracrine mode of action. In the tertiary follicles, the IGF‐1 staining was absent in oocytes but not in granulosa cells, whereas oocytes showed moderate IGF‐1R staining, suggesting the prevalence of paracrine signalling at ovulation. Finally IGF‐1 and IGF‐1R staining was also observed in granulosa cell cords derived from the atretic follicles with similar immunohistochemical traits as seen in the granulosa cells. This may be an indication of their origin (Akihara et al., 2007).

The IGF‐1 and IGF‐1R staining was more intense in follicles than in luteal cells. This indicates that IGF‐1 and its receptor were present mainly during the follicular phase, and could act at various levels. First, increasing the number of LH receptors on dog granulosa cell (Chastant‐Maillard et al., 2011). Second, increasing the expression of StAR protein and, therefore, the P4 production (Adashi, Resnick, Hernandez, Svoboda, & Van Wyk, 1988; Chen et al., 1997; Devoto et al., 1995; Sekar et al., 2000), which has a positive feedback on the LH surge (Concannon et al., 1975). Third, due to its proliferative and angiogenic effects (Grazul‐Bilska et al., 2003), IGF‐1 could be involved not only in ovulation but also in the repair of postovulatory wounds. This is supported potentially in the present study, as IGF‐1 was detected in the ovarian surface epithelium (the layer of squamous to cuboidal cells that surrounds the ovary). Cortical tubules, within the ovarian stroma, were also positive for IGF‐1 and IGF‐1R. These can be the result of the entrapment of the ovarian surface epithelium.

Other stroma cells that stained for IGF‐1 and IGF‐1R were the spindle‐shaped cells. Even though the IGF‐1R staining was weak, indicating a low number of receptors, the high number of spindle‐shaped cells present in the large volume of the stroma, can gather a large number of receptors. Therefore, IGF‐1 could play an important role in the paracrine regulation of the ovarian function.

Our findings showed expression of IGF‐1 and IGF‐1R in the corpus luteum (CL) of the bitch. These results agree with the studies performed in dogs by Balogh el at., (2018) and in other species, such as cows (Schams, Berisha, Kosmann, & Amselgruber, 2002) and pigs (Gadsby, Lovdal, Samaras, Barber, & Hammond, 1996). IGF‐1 and IGF‐1R were expressed in the granulosa lutein cells (GLC) of the non‐pregnant corpus luteum of the bitch. These cells had the appearance of the steroid‐producing cells, with the pale cytoplasm indicating the presence of lipid droplets. The autocrine action of IGF‐1 in the GLC potenciates, their steroidogenic, proliferative and survival capacity of these cells (Bencomo et al., 2006; Pescador, Stocco, & Murphy, 1999). However on the other hand, the theca lutein cells (TLC), which were smaller and more peripheral, showed no detectable or very weak staining and they did not show nuclear positive staining for either IGF‐1 or IGF‐1R.

In all the positively stained cells, the IGF‐1 was located in the nuclei and weakly in the cytoplasm, while IGF‐1R was detected mainly in the cytoplasm, and to a lower degree, in the nuclei. These staining patterns agree with those found in human tumour cells (Matsubara et al., 2016). The cytoplasm localization of IGF‐1 and IGF‐1R may be partly due to de novo synthesis in the cytoplasm. The nuclear localization of IGF‐1 and IGF‐1R localization supports previous studies that have demonstrated that IGF‐1‐mediated IGF‐1R internalization occurs to the nucleus, where it can regulate transcription (Oberbauer, 2013). It has been suggested that the localization varies according the physiological condition of the individual (Oberbauer, 2013; Ohtsuki et al., 2005). In humans, it has been shown that under the influence of oestrogens, an IGF‐1 isoform (IGF‐1b), which contains a nuclear localization signal at the C‐terminus, is expressed and may facilitate IGF‐1 receptor transportation to the nucleus (Oberbauer, 2013; Shavlakadze, Winn, Rosenthal, & Grounds, 2005). The presence of nuclear IGF‐1R was initially described in human tumour cells and no malignant tissues characterized by a high proliferation rate (Aleksic et al., 2010). However, a recent study has shown that the nuclear IGF‐1R is not restricted to tumour cells but it is also present in normal cells (Solomon‐Zemler et al., 2017). The intracellular localization of IGF‐1 and its receptor may constitute a potential regulatory mechanism of the ovarian function in the bitch.

### 4.3 Conclusions

The immunolocalization of IGF‐1 and IGF‐1R in the ovary of the bitch showed a wide distribution, suggesting that this growth factor could be involved in many functions such as follicular development, ovulation, postovulatory wound healing and corpus luteum maintenance. Its localization at different stages of follicular development suggests a change in its mode of action from autocrine to paracrine as the follicle develops. The cytoplasmic and nuclear localization of both IGF‐1 and IGF‐1R reflect the complexity of the IGF‐1 signalling. Further studies are required to shed light on the biological and clinical relevance of the IGF‐1 system in the reproductive physiology of the bitch. Under the conditions of the current study, the IGF‐1 had no effect on the in vitro nuclear maturation of canine oocytes.

## AUTHOR CONTRIBUTION

Diogo José Cardilli: Data curation; Investigation; Methodology. Kellen Sousa Oliveira: Conceptualization; Investigation; Methodology; Validation; Visualization. Carolina Franchi‐João: Conceptualization; Formal analysis; Investigation; Methodology. Faviana Azevedo‐Voorwald: Conceptualization; Formal analysis; Investigation; Methodology. Marco Augusto Machado‐Silva: Investigation; Methodology. João Ademir Oliveira: Investigation; Methodology. Maria‐Jesus Sánchez Calabuig: Validation; Writing‐original draft; Writing‐review & editing. Gilson Toniollo: Validation; Visualization; Writing‐review & editing. José Félix Pérez‐Gutiérrez: Supervision; Validation; Visualization; Writing‐original draft; Writing‐review & editing.

### PEER REVIEW

The peer review history for this article is available at https://publons.com/publon/10.1002/vms3.347.
